# Safety and efficacy of prothrombin complex concentrate as first-line treatment in bleeding after cardiac surgery

**DOI:** 10.1186/s13054-015-1172-6

**Published:** 2016-01-06

**Authors:** Giangiuseppe Cappabianca, Giovanni Mariscalco, Fausto Biancari, Daniele Maselli, Francesca Papesso, Marzia Cottini, Sandro Crosta, Simona Banescu, Aamer B. Ahmed, Cesare Beghi

**Affiliations:** 1 Department of Surgical and Morphological Sciences, Cardiac Surgery Unit, Varese University Hospital, University of Insubria, Varese, Italy; 2 Department of Cardiovascular Sciences, Clinical Sciences Wing, Glenfield Hospital, University of Leicester, Groby Road, Leicester, LE39QP UK; 3 Department of Surgery, Oulu University Hospital, Oulu, Finland; 4 Department of Cardiovascular Surgery, Cardiac Surgery Unit, S.Anna Hospital Catanzaro, Catanzaro, Italy; 5 Cardiac Intensive Care Unit, Varese University Hospital, University of Insubria, Varese, Italy; 6 Department of Anaesthesia and Critical Care, Glenfield Hospital, University Hospitals of Leicester NHS Trust, Leicester, UK

## Abstract

**Background:**

Bleeding after cardiac surgery requiring surgical reexploration and blood component transfusion is associated with increased morbidity and mortality. Although prothrombin complex concentrate (PCC) has been used satisfactorily in bleeding disorders, studies on its efficacy and safety after cardiopulmonary bypass are limited.

**Methods:**

Between January 2005 and December 2013, 3454 consecutive cardiac surgery patients were included in an observational study aimed at investigating the efficacy and safety of PCC as first-line coagulopathy treatment as a replacement for fresh frozen plasma (FFP). Starting in January 2012, PCC was introduced as solely first-line treatment for bleeding following cardiac surgery.

**Results:**

After one-to-one propensity score–matched analysis, 225 pairs of patients receiving PCC (median dose 1500 IU) and FFP (median dose 2 U) were included. The use of PCC was associated with significantly decreased 24-h post-operative blood loss (836 ± 1226 vs. 935 ± 583 ml, *p* < 0.0001). Propensity score–adjusted multivariate analysis showed that PCC was associated with significantly lower risk of red blood cell (RBC) transfusions (odds ratio [OR] 0.50; 95 % confidence interval [CI] 0.31–0.80), decreased amount of RBC units (β unstandardised coefficient −1.42, 95 % CI −2.06 to −0.77) and decreased risk of transfusion of more than 2 RBC units (OR 0.53, 95 % CI 0.38–0.73). Patients receiving PCC had an increased risk of post-operative acute kidney injury (AKI) (OR 1.44, 95 % CI 1.02–2.05) and renal replacement therapy (OR 3.35, 95 % CI 1.13–9.90). Hospital mortality was unaffected by PCC (OR 1.51, 95 % CI 0.84–2.72).

**Conclusions:**

In the cardiac surgery setting, the use of PCC compared with FFP was associated with decreased post-operative blood loss and RBC transfusion requirements. However, PCC administration may be associated with a higher risk of post-operative AKI.

**Electronic supplementary material:**

The online version of this article (doi:10.1186/s13054-015-1172-6) contains supplementary material, which is available to authorized users.

## Background

Bleeding after cardiac surgery requiring surgical reexploration and blood component transfusion is associated with increased morbidity and mortality [1–4]. It is also accompanied by increased rates of late mortality as well as poorer functional outcomes, leading to a substantial morbidity burden and medical costs [1–4]. Therefore, several plasma-derived and recombinant coagulation factors have been tested and introduced for the treatment of excessive bleeding and coagulopathy following cardiac surgery [5–7]. Experimental and clinical studies have recently documented an improved efficacy exerted by prothrombin complex concentrates (PCC) in treating bleeding disorders [8–15]. PCC offers a rapid method for replacing vitamin K–dependent clotting factors and restoring normal haemostasis in the context of over-anticoagulation, being quicker to prepare than fresh frozen plasma (FFP) and allowing administration without warming [16–18]. Its administration also avoids the volume overload usually associated with FFP, reducing the incidence of blood transfusions and the risk of transfusion-related acute lung injury [16–18]. In addition, PCC also has a better safety profile than FFP because of its viral inactivation, minimising the risk of transmission of a variety of infective agents, including prions [16]. However, despite these potential advantages, concerns about an increased risk of thrombogenic events have been raised, and only a minority of studies have investigated the use of PCCs in cardiac surgery. The researchers in the majority of these studies reported the use of PCC to treat severe coagulopathy in high risk patients, to reverse the effect of oral anti-coagulants or even to evaluate the effect of PCC on cardiopulmonary bypass (CPB)–induced coagulopathy in experimental models [7–15]. The purpose of the present study, therefore, was to investigate the safety and efficacy of PCC as first-line treatment in coagulopathy in a consecutive series of patients undergoing cardiac surgery.

## Methods

### Population

Between January 2005 and December 2013, all consecutive patients undergoing isolated coronary artery bypass graft (CABG), valve surgery (with or without concomitant CABG) and proximal aortic procedures at Varese University Hospital were included in this observational study. Elective, urgent or emergency procedures were all included. Off-pump CABG procedures, along with other cardiac operations (cardiac tumour removal, left ventricular remodelling, adult congenital cardiac operation, post-infarction ventricular septal defect and free wall rupture repairs), were not included. Patients who died intra-operatively without blood product administration were also excluded from the analysis. All data were prospectively collected and recorded in computerised database registries that remained consistent over the study period [4, 19, 20]. Information about demographics, co-morbidities, medical and surgical history, operative details and post-operative events during the hospital stay were all registered. The study protocol was approved by the local institutional review board (Comitato Etico Provinciale di Varese s.n. 07/04/2015). Patient consent was waived due to the retrospective and observational nature of the study.

### Patient management

Pre-operative management, anaesthetic and surgical techniques were standardised for all patients and have been reported previously [4, 19, 20]. Generally, anti-platelet drugs and anti-coagulation drugs were discontinued on the day of hospitalisation (median 2 and 3 days before surgery, respectively). Other medications were routinely omitted on the day of the operation and restarted on the first post-operative day, unless clinically contraindicated. All surgical procedures were performed through a median sternotomy approach, and CPB was undertaken in standardised fashion, with cannulation of the ascending aorta and right atrial or bicaval venous cannulation. Intravenous heparin was dosed as 300 IU/kg body weight, and the activated clotting time (ACT) was maintained above 450 seconds. In addition, in all these cases, tranexamic acid was intravenously administered after the induction of anaesthesia until the end of the operation (20 mg/kg for the first hour and 2 mg/kg thereafter). After the CPB, the circulating heparin was antagonised with protamine sulphate at a ratio of 1 mg of protamine per 100 IU of heparin. A prolonged ACT after surgery was treated with an additional dose of protamine sulphate. Generally, when visual inspection revealed microvascular bleeding or an important blood collection in the cardiotomy reservoir (generally 200 ml after weaning from CPB), the patient received an assessment of the haemostasis/coagulation profile through thromboelastography (TEG; Haemoscope, Niles, IL) with and without heparinase. Therefore, peri-operative need for blood products, including FFP and platelets, was determined on an individual basis. Transfusion was guided by point-of-care thromboelastography, prothrombin and activated partial thromboplastin times, and platelet count. Homologous red blood cells (RBC) were intra-operatively administered to maintain the haemoglobin concentration >7 g/dl or the haematocrit higher than 20 % during CPB, whilst they were post-operatively given when haemoglobin was <8 g/dl. Platelets were transfused when their count was ≤60 × 10^9^/L. Additional blood product transfusions, however, were done at the discretion of the individual surgeon or attending anaesthesiologist. Aprotinin or other haemostatic agents were not used in this series. At the end of surgery, patients were transferred to the intensive care unit (ICU) and managed according to the unit protocols [4, 19, 20].

### Outcome end-points and definitions

The primary end-point was the impact of PCC on hospital mortality. Secondary end-points were the effect of PCC on post-operative complications and blood transfusion products.

Patients receiving PCC constituted the study group, and PCC was administered intra-operatively before chest closure or in the ICU within the first post-operative hours. Uman Complex D.I. (Kedrion; Castelvecchio Pascoli, Italy) was the sole PCC available in our institution, and starting in January 2012 it constituted the first-line therapy in coagulation management, replacing FFP. PCC contains clotting factors II, IX and X and is subjected to two steps of viral inactivation—first, solvent/detergent treatment and then heat treatment (100 °C for 30 minutes)—and is supplied as 500 IU of factor IX (20 ml) in vials [21]. Other analysed variables were defined as previously described [4, 19, 20]. Generally, blood product transfusions were counted by units. Haemorrhagic complications accounted for the need for reexploration for bleeding or cardiac tamponade. Generally, significant post-operative bleeding requiring surgical reexploration was defined as >300 ml during the first hour, >250 ml during the second hour, >200 ml during the third hour or a total of 1000 ml or more during the first 6 h. However, the decision regarding haemorrhagic reexploration was made by the surgeon in charge. Chest tube outputs were used as a measure of blood loss, and our analysis was based on the total volume of loss during the first 24 h of the patient’s stay in the ICU. Post-operative acute kidney injury (AKI) was defined according to the consensus RIFLE criteria (risk, injury, failure, loss of function, and end-stage renal disease) using the maximal change in serum creatinine and estimated glomerular filtration rate during the first 7 post-operative days compared with pre-operative baseline values [22].

### Statistical analysis

Clinical data were prospectively recorded and tabulated using Microsoft Excel software (Microsoft, Redmond, WA, USA). Continuous data are reported as mean and standard deviation or median and interquartile range (IQR), as appropriate. Nominal variables were reported as counts and percentages. Fisher’s exact test, χ^2^ test and the Mann-Whitney *U* test were used for univariate analysis. No attempt to replace missing values was made. Multivariate analysis was performed using logistic and linear regression. The area under the receiver operating characteristic (ROC) curve was used to represent the regression probabilities.

**Fig. 1:**
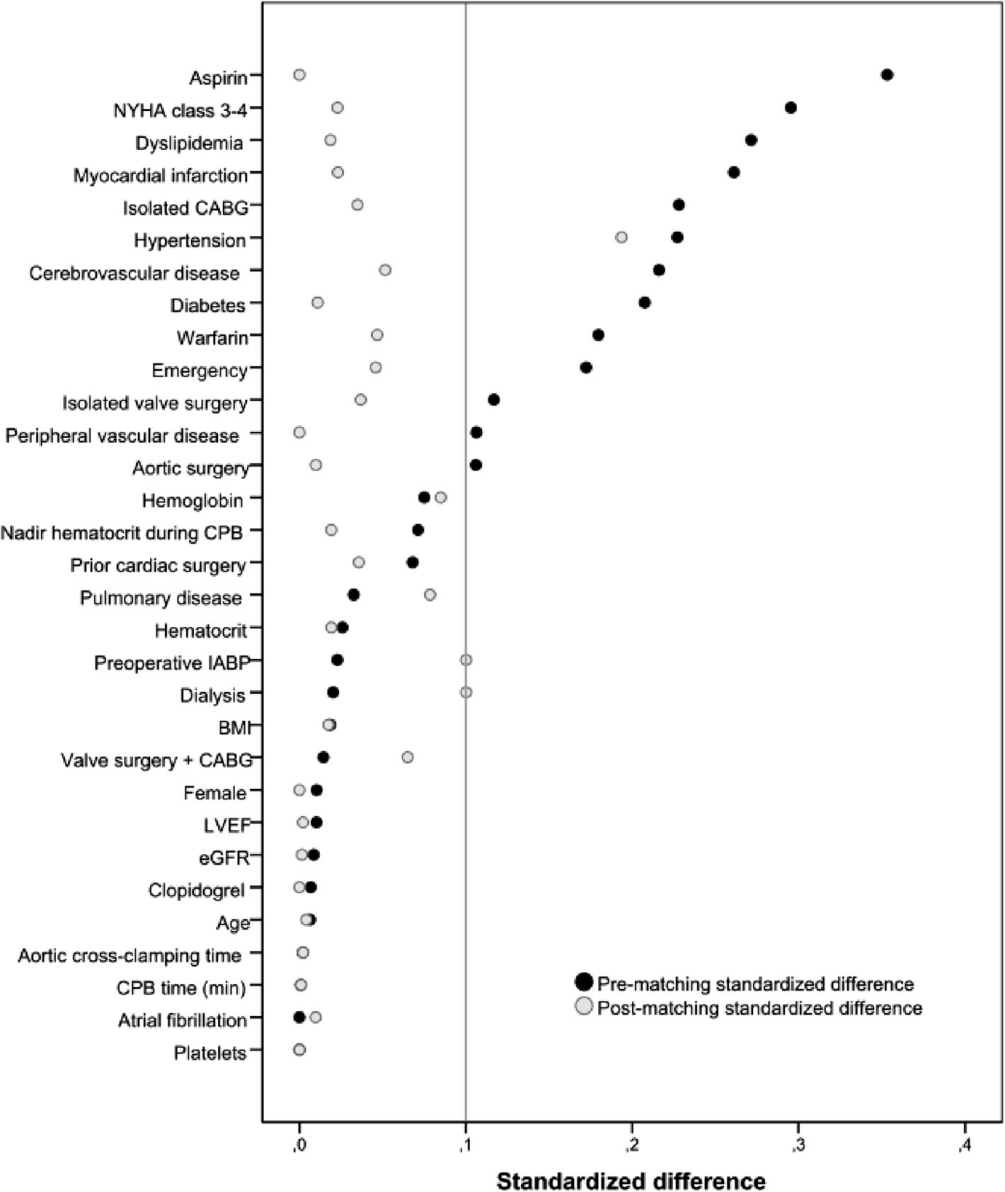
Standardized differences before and after propensity score matching comparing covariate values for patients with or without PCC administration

Since January 2012, PCC has been used systematically as first-line therapy in coagulation management, completely replacing FFP, and this PCC use was consistently followed. Therefore, the PCC study group was matched with the historical series of patients who received FFP before this time point. Because the study groups (i.e., the PCC and the FFP groups) significantly differed in a number of baseline and operative variables, a propensity score was calculated by logistic regression to estimate the probability of being assigned to each of the study treatments. The propensity score was calculated in a non-parsimonious way, including all 31 pre-operative and operative variables listed in Tables 1 and 2. The obtained propensity score was used for adjusted analysis in the overall series and for one-to-one propensity score caliper matching. The caliper width chosen was 0.2 times the standard deviation of the logit of the propensity score (i.e., 0.01). Propensity score was used as a covariate, along with the treatment method, in the multivariate analysis model for each outcome end-point. After the propensity score matching was performed, differences between the two groups were assessed. Absolute standardised differences were estimated to evaluate the pre-match and post-match imbalance, and a standardised difference <0.1 was considered a negligible difference in the mean or prevalence of a covariate between treatment groups (Fig. 1) [23, 24]. Finally, the significance within the models was evaluated with the Wald test, whereas the strength of the association of variables with post-operative outcomes was estimated by calculating the odds ratio (OR), the β unstandardised coefficient and 95 % confidence intervals (CIs). The model was calibrated using the Hosmer-Lemeshow goodness-of-fit test, as well as residual diagnostics (deviance and degrees of freedom of β values). Model discrimination was evaluated by using the area under the ROC curve. All tests were two-sided with the α level set at 0.05 for statistical significance. Statistical analysis was performed using IBM SPSS version 22.0 software (IBM, Armonk, NY, USA).

**Table 1:** Baseline characteristics of patients with reference to PCC administration

	Overall series				Propensity score–matched pairs			
	No PCC (*n* = 680)	PCC (*n* = 291)	*p* Value	Pre-match SD	No PCC (*n* = 225)	PCC (*n* = 225)	*p* Value	Post-match SD
Age, yr	69.8 ± 10.1	69.0 ± 12.3	0.963	0.006	69.7 ± 10.6	69.2 ± 11.6	0.932	0.004
Female sex	263 (38.7)	114 (39.2)	0.884	0.01	91 (40.4)	91 (40.4)	1.000	0
BMI, kg/m^2^	25.0 ± 4.5	25.4 ± 4.8	0.258	0.019	25.4 ± 4.9	25.0 ± 4.7	0.400	0.017
eGFR, ml/min/1.73 m^2^	61 ± 21	66 ± 26	0.003	0.009	64 ± 23	64 ± 26	0.878	0.001
Dialysis	14 (2.1)	7 (2.4)	0.734	0.02	3 (1.3)	6 (2.7)	0.503	0.1
Haematocrit, %	38 ± 6	37 ± 5	0.047	0.026	36 ± 6	37 ± 5	0.206	0.019
Haemoglobin, g/dl	12.4 ± 2.0	12.1 ± 1.9	0.002	0.075	11.8 ± 2.0	12.1 ± 1.9	0.128	0.085
Platelets, 10^9^/L	210 ± 83	213 ± 79	0.552	0	216 ± 88	209 ± 74	0.418	0
Aspirin	279 (39.9)	69 (23.7)	<0.0001	0.353	60 (26.7)	60 (26.7)	1.000	0
Clopidogrel	64 (9.4)	28 (9.6)	0.918	0.007	22 (9.8)	22 (9.8)	1.000	0
Warfarin	94 (13.8)	24 (8.2)	0.015	0.18	24 (10.7)	21 (9.3)	0.637	0.047
NYHA class III-IV	94 (13.8)	74 (25.4)	<0.0001	0.295	42 (18.7)	44 (19.6)	0.810	0.023
Atrial fibrillation	145 (21.3)	62 (21.3)	0.995	0	49 (21.8)	50 (22.2)	0.909	0.01
Hypertension	379 (55.7)	194 (66.7)	0.002	0.227	143 (53.6)	142 (63.1)	0.922	0.194
Diabetes	152 (22.4)	42 (14.4)	0.005	0.208	37 (16.4)	36 (16.0)	0.898	0.011
Pulmonary disease	86 (12.6)	40 (13.7)	0.641	0.033	34 (15.1)	28 (12.4)	0.412	0.078
Dyslipidaemia	200 (29.4)	123 (42.3)	<0.0001	0.271	85 (37.8)	83 (36.9)	0.845	0.019
Peripheral vascular disease	110 (16.2)	59 (20.3)	0.123	0.106	38 (16.9)	38 (16.9)	1.000	0
Cerebrovascular disease	63 (9.3)	48 (16.5)	0.001	0.216	26 (11.6)	30 (13.3)	0.568	0.052
Prior cardiac surgery	91 (13.4)	46 (15.8)	0.320	0.068	37 (16.4)	34 (15.1)	0.698	0.036
Myocardial infarction	185 (27.2)	48 (16.5)	<0.0001	0.261	43 (19.1)	41 (18.2)	0.809	0.023
LVEF, %	52 ± 13	53 ± 11	0.106	0.01	53 ± 12	52 ± 11	0.796	0.002
Emergency	161 (23.7)	49 (16.8)	0.018	0.172	45 (20.0)	41 (18.2)	0.632	0.046
Pre-operative IABP	36 (5.3)	14 (4.8)	0.755	0.023	14 (6.2)	9 (4.0)	0.284	0.1

*BMI* body mass index, *eGFR* estimated glomerular filtration rate, *IABP* intra-aortic balloon pump, *LVEF* left ventricular ejection fraction, *NYHA* New York Heart Association, *PCC* prothrombin complex concentrates, *SD* standard deviation

Continuous variables are reported as mean and standard deviation; nominal variables are reported as counts (percentages)

**Table 2:** Operative data of patients with reference to PCC administration

	Overall series				Propensity score–matched pairs			
	No PCC (*n* = 680)	PCC (*n* = 291)	*p* Value	Pre-match SD	No PCC (*n* = 225)	PCC (*n* = 225)	*p* Value	Post-match SD
Type of surgery			0.009				0.901	
Isolated CABG	173 (25.4)	47 (16.2)		0.228	39 (17.3)	36 (16.0)		0.035
Isolated valve surgery	226 (33.2)	113 (38.8)		0.117	92 (40.9)	88 (39.1)		0.037
Valve surgery + CABG	154 (22.6)	64 (22.0)		0.014	47 (20.9)	53 (23.6)		0.065
Aortic surgery	127 (18.7)	67 (23.0)		0.106	47 (20.9)	48 (21.3)		0.01
CPB time, min	152 ± 64	148 ± 64	0.234	0.001	150 ± 65	152 ± 68	0.734	0.001
ACC time, min	111 ± 51	106 ± 49	0.157	0.002	104 ± 46	109 ± 50	0.410	0.002
Nadir Hct during CPB, %	22.9 ± 3.9	23.9 ± 3.6	<0.0001	0.071	23.4 ± 4.3	23.7 ± 3.7	0.305	0.019

*ACC* aortic cross-clamp, *CABG* coronary artery bypass graft, *CPB* cardiopulmonary bypass, *Hct* haematocrit, *PCC* prothrombin complex concentrates, *SD* standard deviation

Continuous values are reported as mean and standard deviation; nominal variables are reported as counts and (percentages)

## Results

### Study population

Among the 3454 included patients (mean age 68.0 ± 10.8 years, 32.6 % females), 234 (6.8 %) received isolated PCC, 680 (19.7 %) received isolated FFP and 57 (19.6 %) were administered PCC with concomitant FFP. PCC subjects received a median of 1500 IU (IQR 1500–1500 IU), whereas only 38 (13.1 %) had >1500 IU of PCC. The FFP group received a median of 2 FFP U (IQR 2–4 U). In the overall population, resternotomy for bleeding was necessary in 205 patients (5.9 %) and 2067 (59.8 %) required RBC transfusions. Hospital mortality accounted for 101 patients (2.9 %).

### Group comparisons and outcomes

The two study groups showed significant imbalance in the mean and prevalence of a number of baseline risk factors and operative variables (Table 1). A standardised difference ≥0.1 was pre-operatively observed in 13 of 31 baseline and operative covariates taken into account in this analysis (Fig. 1). Major imbalances were documented in the prevalence of pre-operative use of warfarin and aspirin, New York Heart Association classes III and IV, diabetes, cerebrovascular disease, myocardial infarction, isolated CABG, and emergency procedures. However, the incidence of platelet transfusion was similar between the FFP and PCC groups (53.2 % vs. 58.4 %, *p* = 0.137) (Table 3).

**Table 3:** Post-operative outcomes of patients with reference to PCC administration

	Overall series				Propensity score–matched pairs		
	No PCC (*n* = 680)	PCC (*n* = 291)	*p* Value	Propensity score adjusted (OR, 95 % CI or β^a^, 95 % CI)	No PCC (*n* = 225)	PCC (*n* = 225)	*p* Value
In-hospital mortality	49 (7.2)	24 (8.2)	0.573	1.51, 0.84–2.72	19 (8.4)	21 (9.3)	0.740
Post-operative IABP	81 (11.9)	21 (7.2)	0.029	0.61, 0.35–1.09	32 (14.2)	17 (7.6)	0.023
Inotropes	559 (82.2)	217 (74.6)	0.007	0.74, 0.50–1.08	182 (80.9)	170 (75.6)	0.171
Vasopressors	177 (26.0)	92 (31.6)	0.075	1.18, 0.83–1.67	67 (29.8)	71 (31.6)	0.683
Peri-operative MI	35 (5.1)	14 (4.8)	0.827	1.22, 0.58–2.58	13 (5.8)	12 (5.3)	0.837
Stroke	38 (5.6)	20 (6.9)	0.439	1.19, 0.32–5.21	9 (4.0)	14 (6.2)	0.284
AKI	159 (23.4)	91 (31.3)	0.010	1.44, 1.02–2.05	60 (26.7)	68 (30.2)	0.403
RRT	8 (1.2)	9 (3.1)	0.037	3.35, 1.13–9.90	4 (1.8)	8 (3.6)	0.381
Post-operative AF	338 (49.7)	165 (56.7)	0.046	1.34, 0.97–1.84	124 (55.1)	136 (60.4)	0.252
Resternotomy for bleeding	114 (16.8)	42 (14.4)	0.365	0.95, 0.61–1.47	42 (18.7)	33 (14.7)	0.255
Blood loss, ml	908 ± 625	803 ± 1100	<0.0001	−96.5, −222.1 to −29.0	935 ± 583	836 ± 1226	<0.0001
RBC transfusion	619 (91.0)	243 (83.5)	0.001	0.50, 0.31–0.80	210 (93.3)	189 (84.0)	0.002
RBC transfusion, U	4.9 ± 4.5	3.4 ± 3.0	<0.0001	−1.42, −2.06 to −0.77	5.2 ± 4.3	3.4 ± 3.1	<0.0001
RBC transfusion >2 U	463 (68.1)	154 (52.9)	<0.0001	0.53, 0.38–0.73	158 (70.2)	114 (50.7)	<0.0001
Platelet transfusion	362 (53.2)	170 (58.4)	0.137	1.27, 0.92–1.75	123 (54.7)	133 (59.1)	0.341
Ventilation, h	82 ± 202	68 ± 99	0.160	−6.09, −34.12 to 21.95	73.2 ± 98.7	68 ± 95	0.871
ICU stay, h	125 ± 175	112 ± 122	0.191	−4.34, −1.49 to 0.63	128 ± 152	110 ± 118	0.954
In-hospital stay, days	13.2 ± 12.1	11.4 ± 7.7	0.589	−1.47, −3.20 to 0.27	14.1 ± 12.9	11.4 ± 7.9	0.115

*MI* myocardial infarction, *AF* atrial fibrillation, *AKI* acute kidney injury, *CI* confidence interval, *IABP* intra-aortic balloon pump, *ICU* intensive care unit, *OR* odds ratio, *PCC* prothrombin complex concentrates, *RBC* red blood cells, *RRT* renal replacement therapy

Continuous values are reported as mean and standard deviation; nominal variables are reported as counts (percentages)

^a^β value represents the β unstandardised coefficient of the linear regression analysis

The unadjusted univariate analysis demonstrated that the use of PCC was associated with a significantly lower risk of post-operative need for an intra-aortic balloon pump (*p* = 0.029) and inotropes (*p* = 0.007), as well as with decreased post-operative blood loss (*p* < 0.001) and need for RBC transfusions (overall use: *p* = 0.001; number of units transfused: *p* < 0.001; more than 2 U transfused: *p* < 0.001). However, patients receiving the PCC had an increased risk of post-operative AKI (31.3 % vs. 23.4 %, *p* = 0.010) and renal replacement therapy (RRT) (3.1 % vs. 1.2 %, *p* = 0.037). Multivariate analysis confirmed that the PCC were associated with higher risk of post-operative AKI (OR 1.70, 95 % CI 1.20–2.43, *p* = 0.003) and tended to be associated with a higher risk of RRT (OR 3.35; 95 % CI 0.93–12.14, *p* = 0.065).

One-to-one propensity score–matched analysis resulted in 225 pairs with similar baseline characteristics and operative covariates (Tables 1 and 2). The area under the ROC curve of the estimated propensity score was 0.78 (95 % CI 0.75–0.81, *p* = 0.633 by Hosmer-Lemeshow test). Post-match standardised differences for the measured covariates, except hypertension, were <0.1 (most covariates were <0.05), suggesting substantial covariate balance across groups (Fig. 1). Among these matched pairs, the use of PCC was associated with a significantly decreased post-operative blood loss (mean 836 vs. 935 ml, *p* < 0.0001) as well as lower risk of RBC transfusion (84.0 % vs. 93.3 %, *p* = 0.002), transfusion of more than 2 RBC units (50.7 % vs. 70.2 %, *p* < 0.0001) and decreased amount of RBC units transfused (mean 3.4 vs. 5.2 U, *p* < 0.0001) (Table 3). The PCC group also demonstrated a lower risk of resternotomy for bleeding, but the difference did not reach statistical significance (14.7 % vs. 18.7 %, *p* = 0.255). Among these matched pairs, PCC was not associated with either a higher risk of post-operative AKI (*p* = 0.683) or RRT (*p* = 0.403). The incidence of thrombotic/thromboembolic events such as stroke and transient ischemic attack was also similar between patient groups (6.2 % vs. 4.0 %, *p* = 0.284, and 1.3 % vs. 0.4 %, *p* = 0.336, respectively).

Propensity score–adjusted multivariate analysis showed that PCC was associated with significantly lower risk of RBC transfusions (OR 0.50, 95 % CI 0.31–0.80), decreased amount of RBC units (β, −1.42, 95 % CI −2.06 to −0.77) and decreased risk of transfusion of more than 2 RBC units (OR 0.53, 95 % CI 0.38–0.73). However, patients receiving PCC had an increased risk of post-operative AKI (OR 1.44; 95 % CI 1.02–2.05) and RRT (OR 3.35, 95 % CI 1.13–9.90). No difference between groups was observed with reference to hospital mortality (9.3 % vs. 8.4 %, OR 1.51, 95 % CI 0.84–2.72).

### PCC dosage and outcome

Multivariate analysis including all baseline and operative covariates of 291 patients who received PCC demonstrated that the dose of PCC was not associated with any of the main outcome end-points, other than bleeding and use of blood products. In particular, the dose of PCC was not associated with either post-operative AKI (*p* = 0.424) or RRT (*p* = 0.99).

### Group comparisons in the most recent series

To avoid any bias of including control patients who underwent surgery several years before the introduction of PCC in clinical use, only patients operated on from 2009 to 2013 were considered for further analysis of the early outcome. We estimated a propensity score of this subset of patients, and its area under the ROC curve was 0.72 (95 % CI 0.67–0.77, *p* = 0.176 by Hosmer-Lemeshow test). One-to-one propensity score matching employing a caliper width of 0.04 resulted in 123 pairs with similar baseline characteristics and operative covariates. This analysis showed that patients who received PCC had a similar outcome compared with patients who received FFP (Additional file 1: Table S1). Patients who received PCC had significantly less blood loss and received fewer units of RBC.

## Discussion

The present study demonstrates that the early use of PCC instead of FFP was associated with a significant reduction in blood transfusion requirements. However, PCC use was also related to an increased risk of post-operative AKI.

PCC are a quite heterogeneous group of plasma-derived products containing vitamin K–dependent clotting factors. Some contain only three clotting factors, such as the ones commercially available in the United States and Australia, whereas in Europe four-factor PCC are also available [25]. Three-factor PCCs seem to be less powerful than four-factor PCCs in neutralising the effect of warfarin, probably due to the lack of factor VII [26]. Some of the four-factor PCC are even more effective in correcting the coagulopathy because they contain the activated factor VII, like the factor VIII inhibitor bypassing activity (FEIBA), but these products are routinely reserved for patients with acquired autoimmune factor VIII deficiency and they have only occasionally been used in complex cardiac procedures [13, 25].

The number of indications for PCC use has increased during the last few years. These products, initially developed for the treatment of haemophilia B, now have a specific indication in cardiac surgery only when urgent warfarin reversal is required [27, 28]. In a broader surgical context, the 2013 European Society of Anaesthesiology guidelines confirmed the indication to use PCCs in bleeding patients being given oral anti-coagulants (grade 1B recommendation) and suggested the use PCCs in patients not on warfarin treatment in the presence of an elevated bleeding tendency and prolonged clotting time (grade 2C recommendation) [29].

Since 2012, in agreement with our department of haematology, a three-factor PCC has replaced FFP as first-line treatment of bleeding patients following cardiac surgery. The decision was based on a number of theoretical advantages of PCC over FFP. The PCC seems to be faster and more effective than FFP in controlling bleeding, the concentration of clotting factors in PCC is on average 25 times higher than FFP and PCC has been shown to achieve the normalisation of the international normalised ratio (INR) within 30 minutes of administration [26]. PCC is more readily available because it comes lyophilized, can be quickly reconstituted and administered in the operating theatre or in the ICU, and does not require blood group specificity or defrosting. The volume of PCC is typically less than FFP; therefore, they can also be administered quickly in patients susceptible to volume overload, thereby resulting in less haemodilution. On one hand, FFP is known to carry a significant risk of viral infections and transfusion-related lung injuries, whereas the use of PCC has not been associated with such risks to date [30]. On the other hand, there are documented risks associated with the use of PCC. In two different animal models, PCC administration was associated with an increased risk of thromboembolic complications and disseminated intravascular coagulation [31, 32].

The mortality associated with PCC use is not negligible, The complications described include pulmonary embolism, myocardial and renal infarction, stroke, limb ischemia and deep vein thrombosis, although the overall incidence of thromboembolic complications in patients without haemophilia undergoing emergency reversal of warfarin with PCC was low (3.8 %) [33, 34]. The accumulation of factor II after repeated administration of PCC could be the primary determinant of thrombotic events [30]. Importantly, the risk may differ according to the cause of coagulopathy. For patients requiring reversal of oral anti-coagulants, plasma levels of the coagulation inhibitor anti-thrombin may be normal, meaning that factor II levels can be restored to the normal range without causing an imbalance. In contrast, patients with coagulopathy caused principally by haemodilution and/or consumption are likely to have low levels of anti-thrombin as well as factor II, meaning that administration of PCC (with a great quantity of factor II and very small amounts of anti-thrombin) may cause a pro-thrombotic imbalance.

There is a paucity of high-quality data on the use of PCC in cardiac surgery. PCC seems to mitigate diffuse bleeding following CPB in a porcine model [9]. A randomised comparison of a four-factor PCC versus FFP in patients undergoing cardiac surgery with INR >2.1 showed that PCC achieved normalisation of the INR more quickly and in a higher percentage of patients than FFP and that it was associated with a reduced use of blood products [10]. In two further studies, PCC was used as a second-line treatment in patients with severe coagulopathy after bleeding could not be controlled with FFP [12, 13]. Song et al. [13] successfully used FEIBA in 25 patients with life-threatening bleeding refractory to conventional treatments following complex cardiac procedures. In a non-randomised comparison of 150 patients between a three-factor PCC and recombinant factor VIIa, Tanaka et al. [12] reported the superiority of PCC as a second-line treatment in patients with severe bleeding following complex cardiac surgery. In another study, by Görlinger and colleagues [14], the first-line administration of fibrinogen concentrate and PCC combined with a point-of-care testing protocol was associated with reduced blood transfusion requirements. Arnekian et al. [11] presented the non-randomised comparison of three treatments—four-factor PCC alone, PCC plus FFP or FFP alone—in 77 bleeding patients following cardiac procedures. In that study, a low dose of PCC was the most effective in reducing chest tube drainage, reopening for bleeding and blood product use, and no thromboembolic event was noted. Nevertheless, the sample size of this study was small, and the results were biased by the significant differences among the three groups regarding their pre-operative and intra-operative characteristics [11]. Recently, Ortmann et al. [15], who enrolled 251 consecutive patients undergoing pulmonary endarterectomy surgery, observed that PCC was a valid alternative to FFP in patients with coagulopathy previously treated with warfarin.

Our present study has the largest number of patients currently available in the literature who received a three-factor PCC as first-line treatment for post-operative bleeding following routine cardiac operations. These patients were compared with a series of 680 consecutive patients who had surgery over the previous 7 years at the same institution who received FFP because of bleeding. Propensity score–matched analysis revealed that the use of PCC as a first-line treatment for post-operative coagulopathy was effective in reducing bleeding and the need of RBC transfusions. The incidence of platelet transfusion was not different between the two groups, given the very specific indications to prescribe this product. These results were confirmed by the propensity adjusted multivariate analysis of the entire study population, confirming that the use of PCC had a protective effect on post-operative blood loss and RBC transfusion. Given the broad time frame of the study (2005–2013) and the fact that PCC was adopted only in 2012, a sub-analysis of patients who underwent surgery during a shorter study period was performed to overcome this methodological limitation and confirmed the overall findings of this study. These results support, in practice, all the enunciated theoretical advantages of PCC over FFP, not only in terms of readiness and rapidity of treatment but also because of the significantly higher concentration of clotting factors administered with it when compared with FFP.

The comparison of the two propensity-adjusted populations seems to show that the administration of PCC is also safe. Indeed, no difference in terms of hospital mortality was observed between the two groups. Nevertheless, the incidence of renal complications was significantly higher in the PCC group in the unadjusted comparison and remained higher, although not statistically significant, in the propensity score–matched analysis. However, PCC was an independent predictive factor of AKI (1.4-fold) and RRT (3.35-fold) in the propensity-adjusted multivariate analysis. The possible reasons for this result could be several. There is some evidence of the pro-thrombotic effects of PCC and the risks of thromboembolic events such as stroke and renal thrombosis associated with its use [16, 18]. Despite these reports, it seems unlikely that the underlying mechanism of AKI in our series could be purely thromboembolic, given the absence of significant differences in other thromboembolic complications such as myocardial infarction or stroke. It is possible to hypothesise that volume excess given with FFP could somehow have a protective effect on kidney function and that an exclusive use of PCC over FFP in the context of a bleeding patient could lead to a more hypovolemic balance. The use of vasopressors in the PCC group was slightly higher but statistically significant only in the non-adjusted comparison.

This study has limitations. The primary limitation is its retrospective nature. It is a non-randomised study, being a single-centre observational investigation based on prospectively collected data, and a selection bias may have been present, although we attempted to adjust for baseline differences by assessing the role of PCC while stratifying on the propensity score. Despite this careful modelling approach, immeasurable factors may still exist. Physician bias may have influenced PCC administration, patient selection and dose, a difficulty inevitably shared with other studies on the same subject [8–15]. The decision regarding use of FFP and PCC was predominantly clinical and not necessarily based on thromboelastography findings.

## Conclusions

The results of this retrospective study indicate that, in patients with excessive bleeding after cardiac surgery, the use of PCC compared with FFP is associated with decreased post-operative blood loss and RBC transfusion requirements. However, PCC administration may possibly be associated with higher risk of post-operative AKI. The potential benefits and harms associated with the use of PCC in cardiac surgery should be investigated in a randomised study.

## Key messages

- Use of prothrombin complex concentrate (PCC) compared with fresh frozen plasma is associated with decreased post-operative blood loss and RBC transfusion requirements.
- No increased risks of thrombogenic events were associated with the use of PCC.
- PCC administration may be associated with a higher risk of post-operative AKI.

## Additional file

Additional file 1: Table S1. Immediate outcome of patients undergoing cardiac surgery who received prothrombin complex concentrates or fresh frozen plasma. Analysis included patients operated on between 2009 and 2013 only. (DOC 28 kb)

## Abbreviations

ACC: aortic cross-clamp
ACT: activated clotting time
AF: atrial fibrillation
AKI: acute kidney injury
BMI: body mass index
CABG: coronary artery bypass graft
CI: confidence interval
CPB: cardiopulmonary bypass
eGFR: estimated glomerular filtration rate
FEIBA: factor VIII inhibitor bypassing activity
FFP: fresh frozen plasma
Hct: haematocrit
IABP: intra-aortic balloon pump
ICU: intensive care unit
IQR: interquartile range
INR: international normalised ratio
LVEF: left ventricular ejection fraction
MI: myocardial infarction
NYHA: New York Heart Association
OR: odds ratio
PCC: prothrombin complex concentrate
RBC: red blood cells
ROC: receiver operating characteristic
RRT: renal replacement therapy
