# Supplementary material for: Safety and efficacy of prothrombin complex concentrate as first-line treatment in bleeding after cardiac surgery
Source: Crit Care. 2016 Jan 6;20:5. doi: 10.1186/s13054-015-1172-6 (PMC4702344; doi:10.1186/s13054-015-1172-6)
Supplement: Additional file 1: Table S1. — Immediate outcome of patients undergoing cardiac surgery who received prothrombin complex concentrates or fresh frozen plasma. Analysis included patients operated on between 2009 and 2013 only. (DOC 28 kb) [file 13054_2015_1172_MOESM1_ESM.doc]

**Additional file 1: Table S1**. Immediate outcome of patients undergoing cardiac surgery who received prothrombin complex concentrates or fresh frozen plasma. Analysis included patients operated on from 2009 to 2013 only

Propensity score matched pairs

Outcome end-points No PCC PCC

(n= 123 (n= 123) *P*-value

In-hospital mortality 11 (8.9) 10 (8.1) 0.999

Postoperative IABP 14 (11.4) 9 (7.3) 0.381

Inotropes 102 (82.9) 97 (78.9) 0.517

Vasopressors 26 (21.1) 38 (30.9) 0.110

Perioperative MI 4 (3.3) 9 (7.3) 0.254

Stroke 7 (5.7) 7 (5.7) 0.999

AKI 31 (25.2) 39 (31.7) 0.323

RRT 0 1 (1.6) 0.498

Postoperative AF 71 (57.7) 84 (68.3) 0.113

Resternotomy for bleeding 17 (13.8) 20 (16.3) 0.722

Blood loss (mL) 856±539 767±579 0.033

RBC transfusion 110 (89.4) 107 (87.0) 0.693

RBC transfusion (units) 4.2±3.4 3.3±2.7 0.033

RBC transfusion > 2 units 73 (59.3) 60 (48.8) 0.125

Platelets transfusion 64 (52.0) 74 (60.2) 0.199

Ventilation (hours) 69±94 66±86 0.708

ICU stay (days) 5.0±6.0 4.4±4.2 0.421

In-hospital stay (days) 13.8±10.5 11.8±8.0 0.293

Continuous values are reported as mean and standard deviation; nominal variables are reported as counts (percentages).

*AF* atrial fibrillation, *AKI* acute kidney injury, *IABP* intra-aortic balloon pump, *ICU*  intensive care unit, *MI* myocardial infraction, *PCC* prothrombin complex concentrates, *RBC* red blood cells, *RRT* renal replacement therapy.
